# Association of serum concentrations of irisin and the adipokines adiponectin and leptin with epicardial fat in cardiovascular surgery patients

**DOI:** 10.1371/journal.pone.0201499

**Published:** 2018-08-02

**Authors:** Hiroyuki Kaneda, Toshiaki Nakajima, Akiko Haruyama, Ikuko Shibasaki, Takaaki Hasegawa, Tatsuya Sawaguchi, Toshiyuki Kuwata, Syoutarou Obi, Takuo Arikawa, Masashi Sakuma, Hirohisa Amano, Shigeru Toyoda, Hirotsugu Fukuda, Teruo Inoue

**Affiliations:** 1 Department of Cardiovascular Medicine, Dokkyo Medical University and Heart Center, Dokkyo Medical University Hospital, Tochigi, Japan; 2 Department of Cardiovascular Surgery, Dokkyo Medical University, Dokkyo Medical University Hospital, Tochigi, Japan; Universita degli Studi di Catania, ITALY

## Abstract

Epicardial fat located adjacent to the heart and coronary arteries is associated with increased cardiovascular risk. Irisin is a myokine produced by skeletal muscle after physical exercise, and originally described as a molecule able to promote the browning of white adipose tissue and energy expenditure. In order to decrease cardiovascular risk, it has been proposed as a promising therapeutic target in obesity and type 2 diabetes. We investigated the relationships between serum concentrations of irisin and the adipokines adiponectin and leptin and body fat including epicardial fat in patients undergoing cardiovascular surgery. We obtained serum samples from 93 patients undergoing cardiovascular surgery (age 69.6 (SD 12.8) years, BMI 24.1 ± 4.8 kg/m^2^). Computed tomography (CT) and echocardiographic data were obtained from the routine preoperative examination. Subcutaneous fat area (SFA, cm^2^) and visceral fat area (VFA, cm^2^) near the umbilicus were automatically measured using the standard fat attenuation range. Epicardial fat area (EFA, cm^2^) was measured at the position where the heart became a long axis image with respect to the apex of the heart in the coronal section image. Total body fat mass, body fat percentage, and skeletal muscle volume (SMV) were estimated using bioelectrical impedance analysis (BIA). Serum irisin concentration was measured by enzyme-linked immunosorbent assay, and compared with adiponectin and leptin concentrations. The data were also compared with the clinical biochemical data. EFA was strongly correlated with BMI (*P* = 0.0001), non-HDL-C (*P* = 0.029), TG (*P* = 0.004), body fat mass (*P* = 0.0001), and body fat percentage (*P* = 0.0001). Serum leptin concentration showed a significant positive correlation with BMI (*P* = 0.0001) and TG (*P* = 0.001). Adiponectin, but not irisin, showed a significant negative correlation with BMI (*P* = 0.006) and TG (*P* = 0.001). Serum leptin level had a significant positive correlation with EFA, VFA, and SFA. In contrast, the serum adiponectin level was significantly negatively correlated with EFA, VFA, and SFA. The serum irisin level was also negatively correlated with EFA (r = -0.249, *P* = 0.015), and SFA (r = -0.223, *P* = 0.039), and tended to correlate with VFA (r = -0.198, *P* = 0.067). The serum level of adiponectin was negatively correlated with that of leptin (r = -0.296, *P* = 0.012), but there were no significant correlations between irisin and either adiponectin or leptin. Multivariate linear regression demonstrated that EFA showed a positive association with serum leptin level (β = 0.438, *P* = 0.0001) and a negative correlation with serum irisin level (β = -0.204, *P* = 0.038) and serum adiponectin level (β = -0.260, *P* = 0.015) after adjusting for age, sex, and BMI. The present study provided the first evidence of associations of the serum irisin and adipokines (adiponectin and leptin) concentrations with epicardial fat in cardiovascular surgery patients. Irisin may play a role in preventing excess adiposity including epicardial fat, and consequently cardiovascular risk in patients.

## Introduction

Adipose tissue is not only a lipid storage unit, but it also functions as a paracrine and endocrine organ, secreting a number of adipocytokines such as leptin, adiponectin and tumor necrosis factor-α (TNF-α), which have proinflammatory, atherogenic, or protective effects, and contribute to unfavorable metabolic and cardiovascular risk factors [1,2]. An increase in visceral adiposity is associated with high cardiometabolic risk (defined as metabolic syndrome), and increased risks of coronary heart disease (CAD) and type 2 diabetes mellitus (DM). Extra-abdominal fat deposits including epicardial adipose tissue (EAT) as well as intra-abdominal visceral adiposity are now considered as markers of cardiovascular risk [3–5]. In particular, EAT, which interacts locally with the myocardium and coronary arteries, is a metabolically active organ that has a high rate of secretion of inflammatory adipokines such as TNF-α [1,4]. It is also an important source of adiponectin, an anti-inflammatory and anti-atherogenic adipokine. Secretion of adiponectin from EAT can alter adiponectin levels in the systemic circulation [5–7]. Thus, EAT and adipokines released from its adipose tissue play an important role in diseases such as obesity, metabolic syndrome, and consequently cardiovascular diseases, including CAD [8].

Epicardial, subcutaneous, and visceral fat can be measured using simple echocardiography, magnetic resonance imaging (MRI), and multi-slice computed tomography (MSCT) [9,10]. There have been several reports of its clinical associations, especially with CAD [11]. Lima-Martinez et al. [12] showed an inverse proportional relationship between EAT thickness and circulating adiponectin concentration. Similarly, an inverse relationship between adiponectin level and visceral adipose volume measured by computed tomography (CT) has been reported [13], while Harada et al. [14] failed to find a significant association between epicardial fat volume and plasma levels of adiponectin.

On the other hand, irisin is a novel hormone secreted by myocytes (a myokine), that has been proposed to mediate the beneficial effects of exercise on metabolism. Irisin, which is regulated by peroxisome proliferator-activated receptor-γ coactivator-1 (PGC1)-α, is proteolytically cleaved from the product of the FNDC5 gene prior to being released into the circulation [15]. It causes the transformation of white adipocytes into beige / brite adipocytes, white adipocytes with a phenotype similar to brown adipocytes, and then thermogenesis by increasing uncoupling protein 1 (UCP1) levels, and increases energy expenditure in mice and humans [15–17]. In human adipocytes, Huh et al. [18] also showed that irisin induced UCP-1 and consequently increased adipocyte energy expenditure, expression of metabolic enzymes and metabolite intermediates, resulting in inhibition of lipid accumulation. Thus, taken that formation of beige / brite fat has shown to exert anti-obesity and anti-diabetic effects in murine models [19] and humans [20], irisin has been proposed as a potentially attractive therapeutic target for metabolic disorders. In fact, it has been reported that exercises such as aerobic or resistance exercise increase circulating irisin and decrease fat volume in humans [21–23]. However, there have been conflicting results showing a lack of effect of exercise on circulating irisin [24]. Furthermore, in contrast with the anti-obesity and metabolic effects of irisin [21–23], irisin has been reported to be involved in the pathogenesis of various complications of obesity, including dyslipidemia, type 2 DM, and metabolic syndrome [25,26]. We have also reported that the circulating irisin concentration is significantly correlated with HOMA-IR in Japanese obese patients [27]. Thus, it remains uncertain whether irisin exerts anti-obesity effects and decreases adiposity in humans.

Therefore, we investigated the relationship between serum irisin concentration and body fat including epicardial fat in patients undergoing cardiovascular surgery, and compared it with adiponectin and leptin, originally known as adipokines [28,29].

## Materials and methods

### Participants

From October 2015 to December 2016, we evaluated 93 patients undergoing cardiovascular surgery at Dokkyo Medical Hospital. The proposal was approved by the Regional Ethics Committee of Dokkyo Medical University Hospital. The baseline characteristics of the patients are summarized in Table 1. Fifty-eight were men (62%) and 35 were women (38%). The mean age was 69.6 ± 12.8 years, and body mass index (BMI) was 24.1 ± 4.8 kg/m^2^. We assessed the co-incidence of conventional risk factors such as hypertension (HT), diabetes (DM), hyperlipidemia (HL), current smoking, and hemodialysis (HD). Seventy-six patients had HT, 30 had DM, and 41 had HL. Eleven patients were current smokers, and eleven received HD. Forty-two had coronary artery disease (9 patients, one-vessel disease; 4, two-vessel disease, and 29, three-vessel disease). Preoperative functional status was recorded with New York Heart Association (NYHA) classifications with a mean value of 2.1 ± 1.1. Twenty-eight patients underwent coronary artery bypass graft (CABG), and 22 had aortic valve replacement (AVR) or aortic valve implantation (TAVI). Eleven patients had other valve replacement or repair (mitral valve replacement or repair, tricuspid valve repair, replacement or repair). Fourteen patients received the combination of CABG and valve replacement or repair. Twelve patients had aortic surgery, such as endovascular aneurysm repair (EVAR) and artificial blood vessel replacement.

**Table 1:** Patient characteristics.

Total number of patients	93
Male : Female	58 : 35
Age, y	69.6 ± 12.8
BMI, kg/m^2^	24.1 ± 4.8
Risk factors (number of patients)	
Hypertension	76
Diabetes	30
Dyslipidemia	41
Smoking	11
Hemodialysis	11
NYHA	2.1 ± 1.1
Coronary artery disease (number of patients)	42
0-vessel disease	51
1-vessel disease	9
2-vessel disease	4
3-vessel disease	29
Cardiovascular surgery (number of patients)	
CABG	28
AVR or TAVI	22
Other valve replacement / repair	11
Combined	14
Aortic disease (TAR, TEVAR, et al)	12
Others	6
Drugs ((number of patients)	
β-blockers	45
Ca-blockers	31
ACE-I/ARB	48
Diuretics	42
Statin	43
Sulfonylurea	9
α-GI	6
Biguanide	3
DPP4 inhibitor	21
Insulin	8
SGLT2	1
Thiazolidinedione	1

The mean ± SD values are shown.

NYHA, New York Heart Association; CABG, coronary artery bypass grafting; AVR, aortic valve replacement; TAVI, transcatheter aortic valve implantation; TAR, total arch replacement; TEVAR, thoracic endovascular aortic repair; ACE1, angiotensin converting enzyme inhibitor; angiotensin II receptor blocker, ARB; α-glucosidase inhibitor, α-GI: dipeptidyl peptidase-4 inhibitor, DPP-4 inhibitor; sodium glucose cotransporter 2 inhibitor, SGLT2 inhibitor

Fasting venous blood samples were collected into tubes with and without EDTA sodium (1 mg/ml) and into polystyrene tubes without an anticoagulant. Serum and plasma were immediately separated by centrifugation at 3,000 rpm at 4°C for 10 min. Fasting blood sugar (FBS), total cholesterol (T-Chol), hemoglobin A1 (HbA1c), brain natriuretic peptide (BNP), low-density lipoprotein (LDL)-cholesterol (LDL-C), high density lipoprotein (HDL)-cholesterol (HDL-C), non-HDL-C, triglycerides (TG), and estimated glomerular filtration rate (eGFR) were measured before the surgical operation.

eGFR was calculated as follows.

$$\mathrm{Male}:\;eGFR\left(ml/\min/1.73m^{2}\right)=194\left(creatinine^{-1.094}\right)\left(age^{-0.287}\right)$$

$$\mathrm{Female}:\;eGFR\left(ml/\min/1.73m^{2}\right)=0.739\left\{194\left(creatinine^{-1.094}\right)\left(age^{-0.287}\right)\right\}$$

All patients had medical treatment including β-blocking agents (45 patients), Ca channel blockers (31 patients), angiotensin converting enzyme inhibitor (ACE-I) / angiotensin receptor blockers (ARB) (48 patients), diuretics (42 patients), statins (43 patients), and anti-diabetes drugs (sulfonylurea, 9 patients; α-GI, 6 patients; biguanide, 3 patients; DPP4 inhibitor, 21 patients; insulin, 8 patients; sodium glucose cotransporter 2 inhibitor (SGLT2-I) 2, one patient; thiazolidinedione, one patient). Fasting blood glucose (FBS) and biochemical data were analyzed by routine chemical methods at Dokkyo Medical University Hospital clinical laboratory. Levels of the inflammatory marker, high-sensitivity C-reactive protein (hsCRP), were measured by a latex-enhanced nephelometric immunoassay (N Latex CRP II and N Latex SAA, Dade Behring Ltd., Tokyo, Japan). HOMA-IR, an index of insulin resistance, was obtained from the fasting blood insulin (immunoreactive insulin [IRI]) concentration and the FBS level early in the morning, based on the equation:

$$HOMA-IR=\frac{\left(IRI\right)\left(FBS\right)}{405}$$

Oxidative status was studied by measuring hydrogen peroxide (H_2_O_2_) concentration in the serum, in accordance with an automated method (d-ROMs test; Diacron International s.r.l., Grosseto, Italy) using a free radical elective evaluator (F.R.E.E.; Diacron International s.r.l., Grosseto, Italy) as previously described [30]. H_2_O_2_ was converted into radicals that oxidize N, N-diethyl-para-phenylenediamine and can be detected spectrophotometrically using F.R.E.E. The results of the d-ROMs levels were expressed in an arbitrary unit called a Carratelli unit (CARR U), where 1 CARR U corresponds to 0.8 mg/L H_2_O_2_.

### Measurement of a transthoracic echocardiography

Each patient received a pre-operative transthoracic echocardiography (GE Vingmed Ultrasound, Vivid 7Pro, Horten, Norway). Left atrial diameter (LAD), left ventricular (LV) end-diastolic diameter (LVDd), left ventricular end-systolic diameter (LVDs), left ventricular end-diastolic interventricular septum (IVS) thickness (IVST), and left ventricular end-diastolic posterior wall thickness (LVPWT) were measured using M-mode echocardiography. From the apical two- and four-chamber view, left ventricular end-diastolic (LVEDV) and end-systolic volumes (LVESV) were measured using a modified Simpson method and computer-assisted planimetry. Left ventricular ejection fraction (LVEF) was calculated as follows. LV mass was calculated using the Devereux equation:

$$LVEF=\frac{100\left(\mathrm{LVEDV}‑\mathrm{LVESV}\right)}{\begin{array}{l} LVEDV \end{array}}$$

$$LVmass=0.8\left\{1.04{\left(LVDd+IVST+LVPWT\right)}^{3}-LVDd^{3}\right\}+0.6$$

Transmitral peak early diastolic filling (E) and peak atrial contraction (A) wave velocities were measured by using continuous pulse-wave Doppler from the apical windows aligning the ultrasound beam with the transvalvular mitral flow and sample between the tip of the valves, and E/A was calculated.

### Measurements of serum irisin, adiponectin, leptin and TNF-α concentrations

To measure fasting serum irisin, adiponectin, leptin and TNF—α levels, peripheral venous blood was drawn into pyrogen-free tubes without EDTA on the morning of the cardiovascular surgery. Blood samples were promptly centrifuged at 4°C and 3,000 rpm for 10 min, and serum placed in a container for storage at –80°C until performance of an enzyme-linked immunosorbent assay (ELISA) or a Luminex assay. Serum irisin level was determined using an irisin enzyme immunoassay kit (EK-067-29; Phoenix Pharmaceuticals, Burlingame, CA, USA) as previously described [27,31]. Optical density at 450 nm was measured using a microplate reader (Powerscan HT; DS Pharma Biomedical Co. Ltd., Osaka, Japan). The detection threshold was 0.1 ng/ml. Serum adiponectin level was measured by Human Total Adiponectin/Acrp30 Quantikine ELISA Kit (DRP300, R&D Systems, Minneapolis, MN, USA), as described previously [32]. The detection threshold was 0.24 ng/ml. Samples, reagents, and buffers were prepared according to the manufacturers’ manuals. A Luminex assay was applied to determine serum levels of leptin. The serum concentrations of leptin were calculated by comparing the assay readings on a Luminex200™ system (Luminex Co., Austin TX, USA). The detection threshold was 10.2 pg/ml. A Luminex assay was applied to determine serum levels of TNF-α. The serum concentrations of TNF-α were calculated by comparing the assay readings on a Luminex200™ system. The detection threshold was 1.2 pg/ml.

### Measurements of adipose tissue area by computed tomography (CT)

**Fig 1:**
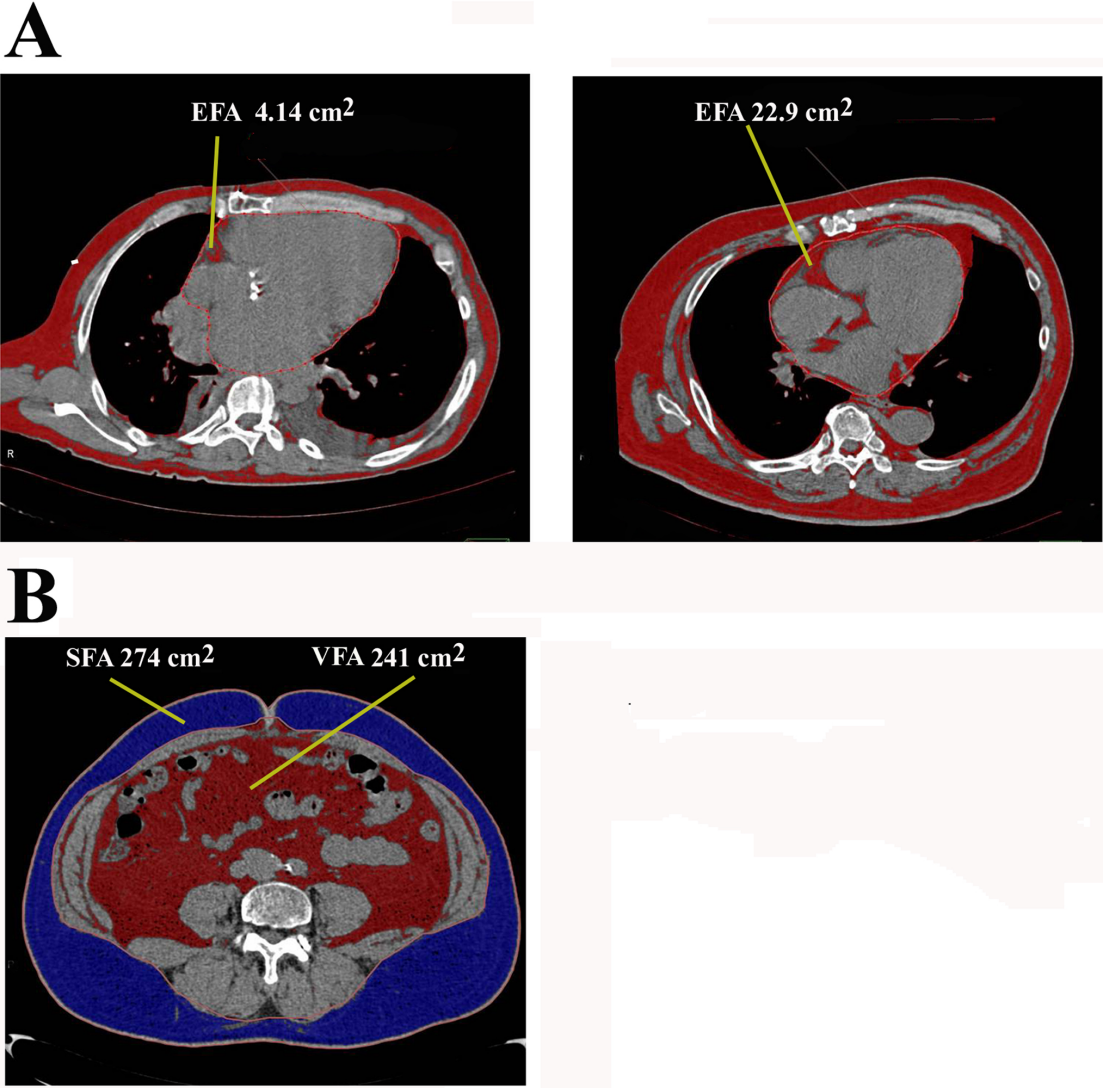
Measurement of fat area by CT scan. A: Measurement of epicardial fat area (EFA). Two representative cases are shown. The image is shown at the position where the heart becomes a long axis image with respect to the apex of the heart in the axial section image with ROI. The heart surrounded by ROI and the epicardial fat area (EFA, cm^2^) was measured by a semi-automated method with CT value defined as -150 to -50 HU. Fat is identified as brown color. EFA is estimated as 4.14 cm^2^ and 22.9 cm^2^, respectively. B: Measurement of subcutaneous and visceral fat area. The areas of abdominal visceral fat (VFA, cm^2^, brown color), and subcutaneous fat (SFA, cm^2^, blue color) were determined at the cross-sectional image of the umbilicus. Fat was measured by a semi-automated method with CT value defined as -150 to -50 HU.

The data of the thoraco-abdominal region in computed tomography (CT) scans were obtained using Ziostation to measure the abdominal visceral, subcutaneous, and epicardial fat as previously reported [33]. The epicardial fat area (EFA, cm^2^) was measured by cardiac fat extraction macro by 3D analysis software in simple chest CT data as shown in Fig 1A. After switching to MPR display with 3D analysis, each section was manipulated so that the heart became a long axis image with respect to the apex of the heart in the axial section image with ROI. The heart surrounded by ROI and EFA (cm^2^) was measured by a semi-automatic method with the CT value defined as -150 to -50 HU. Fig 1A shows two representative cases in cardiovascular patients. The areas (cm^2^) of abdominal visceral and subcutaneous fat were determined at the cross-sectional image of the umbilicus (Fig 1B). If the kidneys and intestinal tract entered the umbilicus, the data at the level near to the umbilicus not including these organs as much as possible were selected. The visceral (VFA, cm^2^) and subcutaneous fat areas (SFA, cm^2^) were measured by a semi-automatic method with the CT value defined as -150 to -50 HU.

### Measurements of the bioelectrical impedance analyzer (BIA)

A multi-frequency bioelectrical impedance analyzer (BIA), InBody S10 Biospace device (Biospacte Co, Ltd, Korea/Model JMW140) was used according to the manufacturer’s guidelines as described in detail previously [34]. BIA estimates body composition using the differences in the conductivity of various tissues due to the differences in their biological characteristics. Conductivity is proportional to water content, and more specifically to electrolytes, and it decreases as the cell approaches a perfect spherical shape. Adipose tissue is composed of round-shaped cells and contains relatively little water compared to other tissues like muscle. Consequently, conductivity decreases as body fat increases. Electrodes are placed at 8 precise tactile-points of the body to achieve a multi-segmental frequency analysis. A total of 30 impedance measurements were obtained using 6 different frequencies (1, 5, 50, 250, 500, and 1000 kHz) at the 5 following segments of the body: right and left arms, trunk, right and left legs. The measurements were carried out while the subjects rested quietly in a supine position, with their elbows extended and relaxed beside their trunk. Body fat mass (BFM), body fat percentage (BF%), lean body mass (LBM), and skeletal muscle volume (SMV) were recorded.

### Statistical analysis

Data are presented as mean value ± SD. The comparison of means between groups was carried out using simple t-test or U-test. Associations among parameters were evaluated using Pearson or Spearman correlation coefficients. Linear regression and multiple linear regression equations were used for multivariate analysis. Leptin, adiponectin, irisin concentrations and EFA were logarithmically transformed to achieve a normal distribution. Multiple linear regression analysis with log (EFA) as the dependent variable was performed to identify independent factors (leptin, adiponectin, and irisin). Age, sex, and BMI were employed as covariates. All analyses were performed using SPSS version 24 (IBM Corp., New York, USA) for Windows. A *P* value of 0.05 was regarded as significant.

## Results

### Characteristics of study patients

The clinical characteristics and sex differences of the study patients are shown in Tables 1 and 2. The mean age of females was higher than that of males (66.8 ± 13.5 years vs. 74.5 ± 9.7 years, *P*<0.01). The TG and T-Chol levels were 111 ± 63 mg/dl, and 168 ± 37 mg/dl, respectively. The LDL-C level was 93 ± 28 mg/dl, and the non-HDL-C level was 114 ± 33 mg/dl. The HDL-C concentration was 52 ± 16 mg/dl. The level of HDL-C in females was higher than in males. The fasting blood glucose (FBS) was 115 ± 30 mg/dl, and HbA1c was 6.2 ± 0.9%. HOMA-IR was 2.35 ± 2.23. The insulin level was significantly lower in females (233 ± 180 μU/mL) than in males (480 ± 686 μU/mL), but HOMA-IR was not different between males and females. The d-ROMs level was 318 ± 86 CARR U, and there were no significant differences in between males and females. In UCG findings, LVDd, LVDs, LV mass, and E/A value in females were significantly lower than in males.

**Table 2:** Sex differences of various parameters.

	Total	Male	Female
number of patients	93	58	35
Age (years)	69.7 (12.6)	66.8 (13.5)	74.5 (9.7)**
BMI, kg/m^2^	24.8 (5.7)	25.0 (6.9)	24.5 (3.1)
NYHA	2.1 (1.1)	2.2 (1.1)	2.1 (0.9)
UCG findings (number of patients)	92	58	34
LAD (mm)	42.4 (9.0)	43.9 (9.9)	40 (6.6)
LVDd (mm)	50.8 (11.2)	54.3 (12.0)	45.1 (6.6)**
LVDs (mm)	35.0 (10.0)	38.1 (10.5)	30.1 (6.7)**
IVST (mm)	10.5 (4.9)	10.2 (5.9)	9.8 (2.3)
LVPWT (mm)	9.8 (3.3)	9.9 (3.8)	9.7 (2.2)
LV mass (g)	178.2 (62.7)	197.8 (63.5)	146.7 (46.4)**
EF (%)	56.1 (11.8)	54.9 (12.5)	58.0 (10.3)
E/A	1.15 (0.90)	1.30 (1.00)	0.91 (0.60)**
CT findings (number of patients)	93	58	35
Epicardial fat area (EPA, cm^2^)	13.8 (9.4)	14.8 (10.3)	11.8 (7.1)
Visceral fat area (VFA, cm^2^)	91.8 (56.3)	103.8 (58.4)	72.0 (45.9)**
Subcutaneous fat area (SFA, cm^2^)	119.4 (73.2)	111.2 (67.3)	132.8 (81.0)
BIA method findings (number of patients)	67	40	27
Body fat mass (BFM, kg)	20.0 (7.6)	19.4 (8.7)	20.7 (5.7)
Body fat percentage (BF%, %)	32.1 (9.3)	28.3 (7.8)	37.6 (8.7)**
Skeletal muscle volume (SMV, kg)	21.2 (5.4)	24.6 (4.2)	16.6 (2.8)**
Lean body mass (LBM, kg)	40.0 (9.5)	45.3 (7.8)	32.1 (5.4)**
Serum level (number of patients)	72	42	30
Leptin, pg/ml	5867 (7316)	4248 (6941)	8134 (7336)**
Adiponectin, ng/ml	8.1 (6.4)	6.95 (5.59)	9.70 (7.22)
Irisin, ng/ml	2.14 (0.55)	2.10 (0.51)	2.21 (0.61)
TNFα, pg/ml	3.48 (2.60)	4.09 (2.88)	2.61 (1.88)*
Biochemical data (number of patients)	93	58	35
TG, mg/dl	111 (63)	102 (52)	126 (76)
T-Chol, mg/dl	168 (37)	159 (35)	183 (34)**
HDL-C, mg/dl	52 (16)	48 (15)	58 (17)**
LDL-C, mg/dl	93 (28)	90 (27)	101 (28)*
Non-HDL-C, mg/dl	114 (33)	110 (29)	122 (39)
BNP, pg/ml	367 (597)	383 (648)	341 (501)
eGFR	59.1 (27.0)	57.3 (29.5)	62.1 (22.0)
HbA1c, %	6.2 (0.9)	6.3 (1.0)	6.0 (0.7)
FBS, mg/dl	115 (30)	118 (34)	110 (18)
hsCRP, mg/dL	0.81 (1.75)	1.08 (2.08)	0.36 (0.77)
d-ROMs (CARR U)	318 (86)	316 (96)	315 (63)
DM parameters (number of patients)	72	42	30
Insulin (I.U./ml)	377 (554)	480 (686)	233 (180)*
HOMA-IR	2.35 (2.23)	3.56 (5.21)	1.21 (1.29)

*P<0.05

**P<0.01.

Male vs. Female TNFα, tumor necrosis factor α; TG, triglyceride; T-Chol, total cholesterol; HDL-C, High density lipoprotein cholesterol; LDL-C, Low density lipoprotein cholesterol; hsCRP, high sensitive C-reactive protein; d-ROMs, derivatives of reactive oxidative metabolites; BNP, brain natriuretic peptide; eGFR, estimate glomerular filtration rate; FBS, Fasting blood sugar; HOMA-IR, Homeostasis model assessment: insulin resistance; UCG, ultrasound cardiogram; LVDd, left ventricular end-diastolic diameter; LVDs, left ventricular end-systolic diameter; IVST, intraventricular septal thickness; LVPWT, left ventricular posterior wall thickness; LVM, left ventricular mass; LVEF, left ventricular ejection fraction; E/A, peak early diastolic transmitral flow velocity / atrial systolic transmitral flow velocity.

The serum leptin level was 5867 ± 7316 pg/ml. It was higher in females than in males (females, 8134 ± 7336 pg/ml; males, 4248 ± 6941 pg/ml, *P*<0.01). The fasting serum adiponectin concentration was 8.1 ± 6.4 ng/ml, and was not significantly different between males and females (males, 6.95 ± 5.59 ng/ml; females, 9.70 ± 7.22 ng/ml). The serum irisin level was 2.14 ± 0.55 ng/ml, and no significant differences between females and males were observed (males, 2.10 ± 0.51 ng/ml; females, 2.21 ± 0.61 ng/ml). The serum TNP-α level was 3.48 ± 2.60 pg/ml. It was higher in males than in females (males, 4.09 ± 2.88 pg/ml; females, 2.61 ± 1.88 pg/ml, *P*<0.05).

Table 2 shows the CT and BIA findings to estimate body composition. The epicardial fat area (EFA) was 13.8 ± 9.4 cm^2^ in all subjects. No significant differences were observed between males and females (males, 14.8 ± 10.3 cm^2^; females 11.8 ± 7.1 cm^2^). The visceral fat area (VFA, cm^2^) was 91.8 ± 56.3 cm^2^ in all subjects. It was significantly higher in males (103.8 ± 58.4 cm^2^) than in females (72.0 ± 45.9 cm^2^, *P*<0.01). By contrast, the subcutaneous fat area (SFA, cm^2^) was not statistically significant between males and females (111.2 ± 67.3 cm^2^ vs. 132.8 ± 81.0 cm^2^). Body fat percentage was 28.3 ± 7.8% in males and 37.6 ± 8.7% in females. It was significantly higher in females (*P*<0.01). On the other hand, skeletal muscle volume, and lean body mass were significantly lower in females, as shown in Table 2.

### Relationships between fat area measured by CT scan and BIA findings and clinical data

First, correlations of the distributed fat tissues were investigated. Epicardial fat area (EFA) was significantly correlated with visceral fat area (VFA, r = 0.797, *P =* 0.0001) and subcutaneous fat area (SFA, r = 0.422, *P* = 0.0001). There was also a significant correlation between VFA and SFA (r = 0.583, *P* = 0.0001).

**Fig 2:**
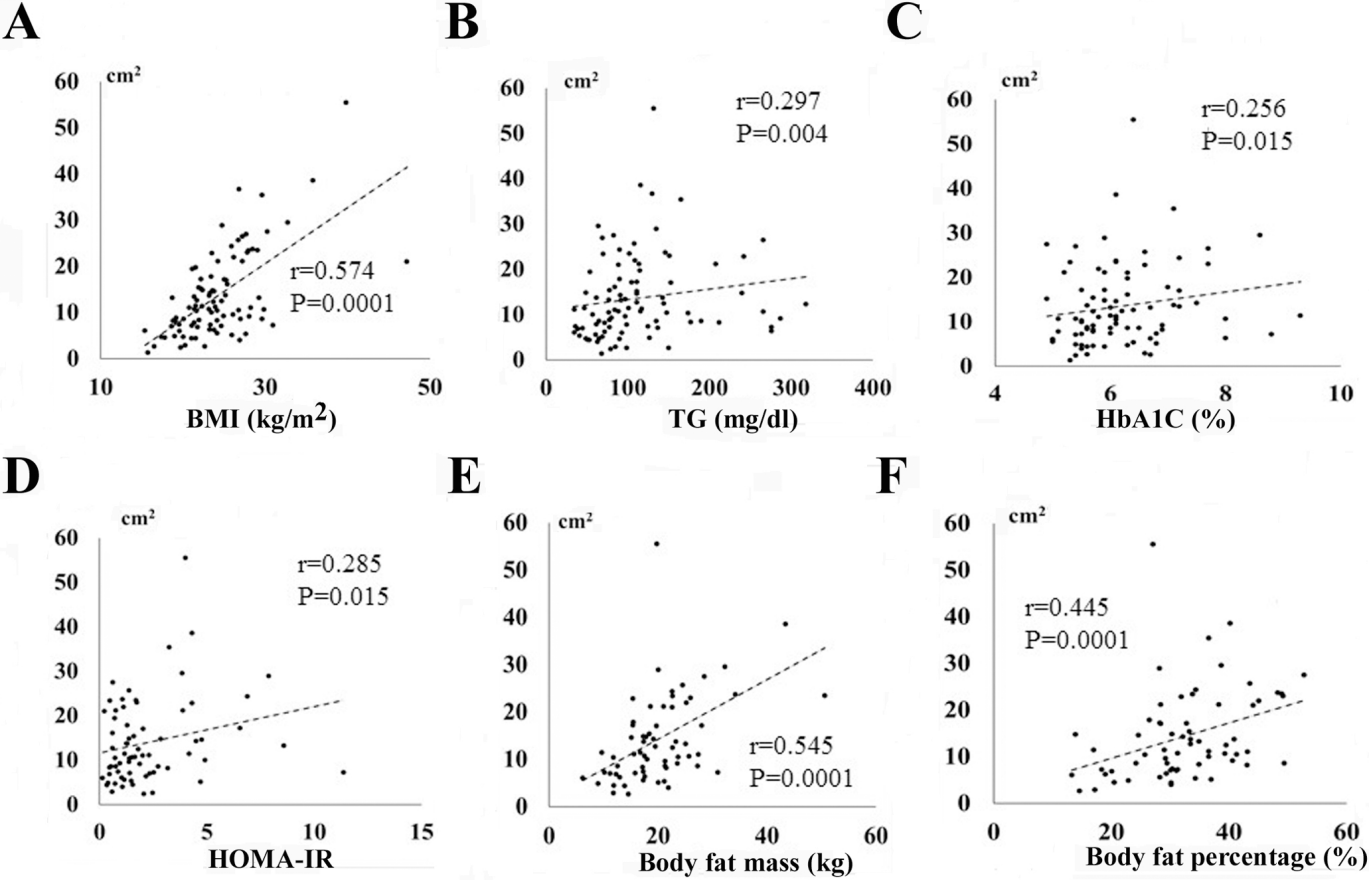
Correlations between epicardial fat area (EFA) and clinical data. Relationship between epicardial fat area (EFA) and BMI (A), triglyceride (TG, B), HbA1C (C), HOMA-IR (D), body fat mass (E), and body fat percentage (F).

Table 3 and Fig 2 show the relationships between clinical data and three parts of fat area (EFA, VFA, and SFA). VFA, but not EFA, was correlated with age (r = -0.254, *P* = 0.018). SFA was significantly negatively correlated with age (r = -0.417, *P* = 0.0001). There were significant positive correlations between BMI and EFA (r = 0.574, *P* = 0.0001, Fig 2A), VFA (r = 0.698, *P* = 0.0001), and SFA (r = 0.644, *P* = 0.0001). On the other hand, there were significant negative correlations between BNP and EFA (r = -0.213, *P* = 0.039), SFA (r = -0.366, *P* = 0.001), and VFA (r = -0.294, *P* = 0.006). EFA was significantly positively correlated with TG (r = 0.297, *P* = 0.004, Fig 2B) and non-HDL-C (r = 0.226, *P* = 0.029). VFA and SFA were positively correlated with TG, T-Chol, LDL-C and non-HDL-C. HbA1C level was positively correlated with EFA (r = 0.256, *P* = 0.015, Fig 2C) and VFA (r = 0.328, *P* = 0.003), but not with SFA (r = 0.078, *P* = 0.487). There was also a positive correlation between EFA and HOMA-IR (r = 0.285, *P* = 0.015, Fig 2D). There were no correlations between d-ROMs and EFA, VFA and SFA (Table 3). Table 3 also shows UCG data and three parts of fat area (EFA, VFA, and SFA). No statistical significant differences were observed in between UCG findings and three parts of fat area (EFA, VFA, and SFA).

**Table 3:** Correlation matrix between fat volume and biochemical data.

CT findings	Epicardial fat area (EFA)	Subcutaneous fat area (SFA)	Visceral fat area (VFA)
Clinical data (number of patients)	93	93	93
Age	-0.092 (0.372)	-0.417 (0.0001**)	-0.254 (0.018*)
BMI	0.574 (0.0001**)	0.698 (0.0001**)	0.644 (0.0001**)
BNP	-0.213 (0.039*)	-0.366 (0.001**)	-0.294 (0.006**)
FBS	0.092 (0.376)	0.033 (0.759)	0.225 (0.036*)
eGFR	-0.062 (0.550)	0.197 (0.069)	-0.008 (0.945)
T-Chol	0.134 (0.201)	0.303 (0.005**)	0.221 (0.042*)
TG	0.297 (0.004**)	0.347 (0.001**)	0.308 (0.004**)
HDL-C	-0.055 (0.596)	-0.2026 (0.811)	-0.003 (0.977)
LDL-C	0.143 (0.170)	0.252 (0.019*)	0.285 (0.008**)
Non-HDL-C	0.226 (0.029*)	0.381 (0.0001**)	0.285 (0.027*)
HbA1C	0.256 (0.015*)	0.078 (0.487)	0.328 (0.003**)
hsCRP	-0.052 (0.616)	-0.169 (0.120)	-0.082 (0.455)
d-ROM	-0.041 (0.699)	-0.035 (0.750)	-0.111 (0.313)
UCG findings (number of patients)	92	92	92
LAD	-0.119 (0.263)	-0.018 (0.870)	-0.010 (0.928)
LVDd	0.037 (0.729)	0.061 (0.579)	0.123 (0.265)
LVDs	-0.037 (0.730)	0.006 (0.961)	0.107 (0.335)
LV mass	0.011 (0.917)	-0.023 (0.836)	0.091 (0.416)
EF	-0.017 (0.870)	0.042 (0.705)	-0.011 (0.923)
E/A	-0.220 (0.058)	0.022 (0.860)	0.050 (0.685)
BIA method (number of patients)	67	67	67
Body fat mass (BFM)	0.545 (0.0001**)	0.640 (0.0001**)	0.578 (0.0001**)
Body fat percentage (BF%)	0.445 (0.0001**)	0.416 (0.0001**)	0.414 (0.0001**)
Skeletal muscle volume (SMV)	0.004 (0.973)	0.078 (0.562)	0.251 (0.057)
Lean body mass (LBM)	0.033 (0.792)	0.114 (0.380)	0.256 (0.047*)
DM parameters (number of patients)	72	72	72
Insulin	0.199 (0.094)	0.233 (0.062)	0.166 (0.185)
HOMA-IR	0.285 (0.015*)	0.161 (0.199)	0.171 (0.174)

*P<0.05

**P<0.01

We also examined the relationships between fat area determined by CT and BIA (Table 3, Fig 2). There were significant positive correlations between body fat mass / body fat percentage and EFA (Fig 2E and 2F), VFA, and SFA.

### Correlation between serum irisin and adipokines (adiponectin, leptin, TNF-α) level and clinical data

**Fig 3:**
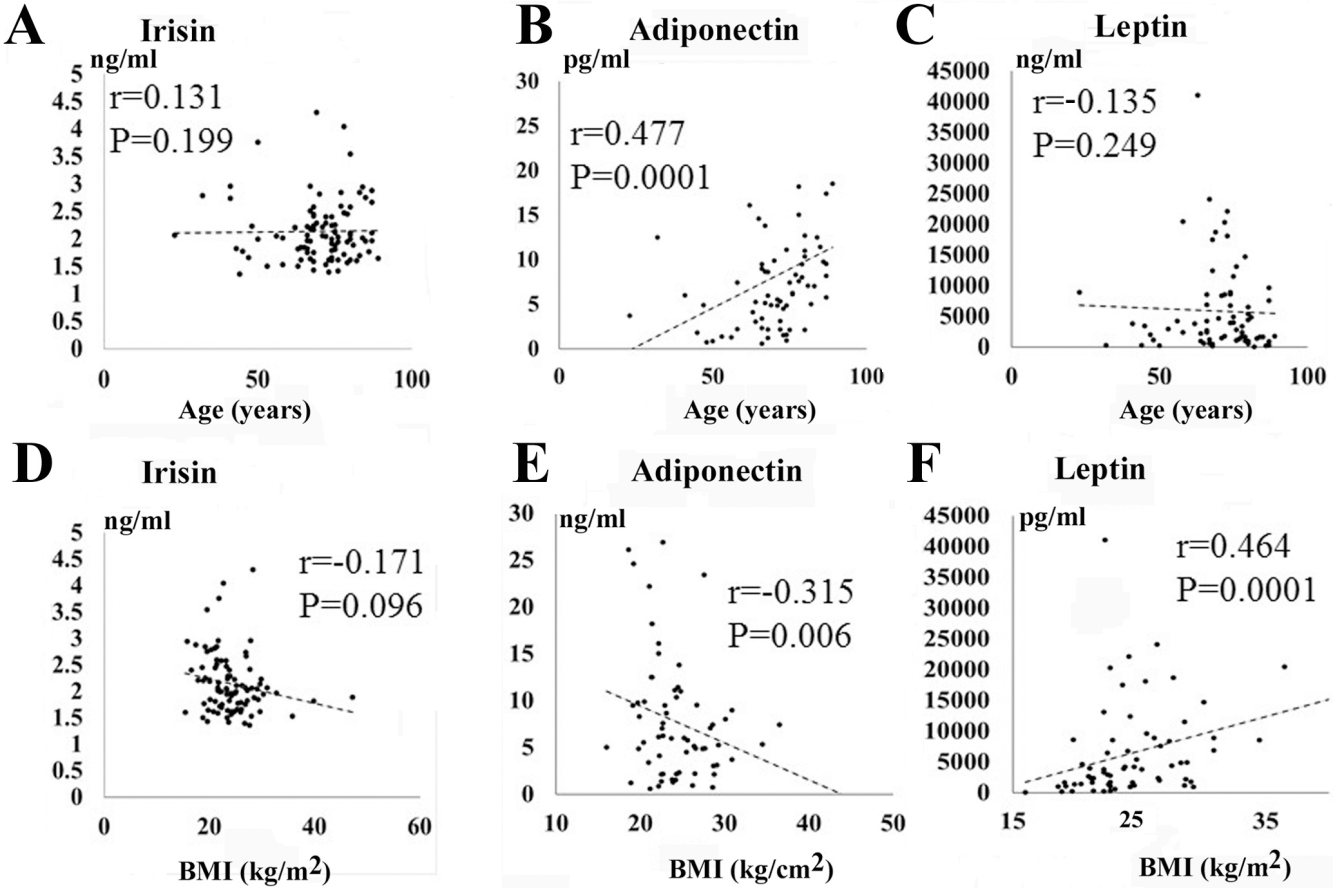
Correlations between serum irisin and adipokines (adiponectin, leptin) concentration and clinical data. A-C: Relationship between age and serum irisin and adipokines level (irisin (A), adiponectin (B), leptin (C)). D-F: Relationship between BMI and serum adipokines level (irisin (D), adiponectin (E), leptin (F)).

The correlations between serum irisin and adipokine (adiponectin, leptin, TNF-α) level and clinical data are shown in Table 4 and Fig 3. The serum irisin and leptin levels were not correlated with age (Fig 3A and 3C), while adiponectin concentration was correlated with age (r = 0.477, *P* = 0.0001, Fig 3B). No statistically significant correlations were found between BMI and serum irisin level (r = -0.171, *P* = 0.096, Fig 3D). However, the serum leptin level was positively correlated with BMI (r = 0.464, *P* = 0.0001, Fig 3F), while the adiponectin level was negatively correlated with BMI (r = -0.315, *P* = 0.006, Fig 3E). The concentration of adiponectin was positively correlated with BNP (r = 0.538, *P* = 0.0001) and HDL-C (r = 0342, *P* = 0.003). It was negatively correlated with FBS (r = -0.267, *P* = 0.021), eGFR (r = -0.243, *P* = 0.037), and TG (r = -0.363, *P* = 0.001). The concentration of TNF-α was positively correlated with BNP (r = 0.336, *P* = 0.003) and negatively correlated with eGFR (r = -0.482, *P* = 0.0001). Similarly, TNF-α and adiponectin level in patients with HD was significantly higher than those without HD, while the leptin and irisin concentration were not significantly different in between patients with HD and those without HD.

**Table 4:** Correlation matrix between clinical data and serum irisin and adipokines (adiponectin and leptin) concentration.

	Irisin	Adiponectin	Leptin
Age	0.131 (0.199)	0.477 (0.0001**)	-0.135 (0.249)
BMI	-0.171 (0.096)	-0.315 (0.006**)	0.464 (0.0001**)
BNP	-0.036 (0.731)	0.538 (0.0001**)	-0.347 (0.002**)
FBS	-0.018 (0.865)	-0.267 (0.021*)	0.103 (0.377)
eGFR	0.178 (0.083)	-0.243 (0.037*)	-0.003 (0.983)
T-Chol	-0.015 (0.888)	-0.013 (0.915)	0.397 (0.0001**)
TG	-0.091 (0.377)	-0.363 (0.001**)	0.423 (0.0001**)
HDL-C	0.051 (0.624)	0.342 (0.003**)	0.035 (0.769)
LDL-C	0.060 (0.560)	0.045 (0.706)	0.271 (0.019*)
Non-HDL-C	-0.035 (0.734)	-0.196 (0.094)	0.411 (0.0001**)
HbA1C	-0.108 (0.309)	-0.219 (0.068)	0.095 (0.433)
hsCRP	0.102 (0.321)	0.024 (0.842)	0.057 (0.628)
d-ROMs	0.112 (0.282)	0.174 (0.147)	-0.049 (0.682)
Insulin	-0.081 (0.487)	-0.202 (0.085)	0.351 (0.002**)
HOMA-IR	-0.0026 (0.822)	-0.145 (0.219)	0.285 (0.013*)
Body fat mass (BFM)	0.098 (0.419)	-0.172 (0.164)	0.491 (0.0001**)
Body fat percentage (BF%)	0.151 (0.212)	0.051 (0.683)	0.444 (0.0001**)
Skeletal muscle volume (SMV)	0.049 (0.645)	-0.266 (0.033*)	-0.297 (0.016*)
Lean body mass (LBM)	0.056 (0.645)	-0.243 (0.047*)	-0.236 (0.052)
Epicardial fat area (EPA)	-0.249 (0.015*)	-0.316 (0.0007**)	0.477 (0.0001**)
Visceral fat area (VFA)	-0.198 (0.067)	-0.430 (0.0001**)	0.416 (0.001**)

*P<0.05

**P<0.01

There were no statistically significant correlations between serum irisin and these clinical data. The concentration of serum leptin was negatively correlated with BNP (r = -0.347, *P* = 0.002), and positively correlated with T-Chol (r = 0.397, *P* = 0.0001), TG (r = 0.423, *P* = 0.0001), LDL-C (r = 0.271, *P* = 0.019), and non-HDL-C (r = 0.411, *P* = 0.0001). The fasting insulin level and HOMA-IR were correlated with serum leptin concentration, but not with the irisin or adiponectin levels. The d-ROMs levels were not statistically correlated with serum irisin and adipokines (adiponectin, leptin) level.

### Correlations between serum irisin and adipokines (TNF-α, adiponectin, leptin) level and BIA findings

**Fig 4:**
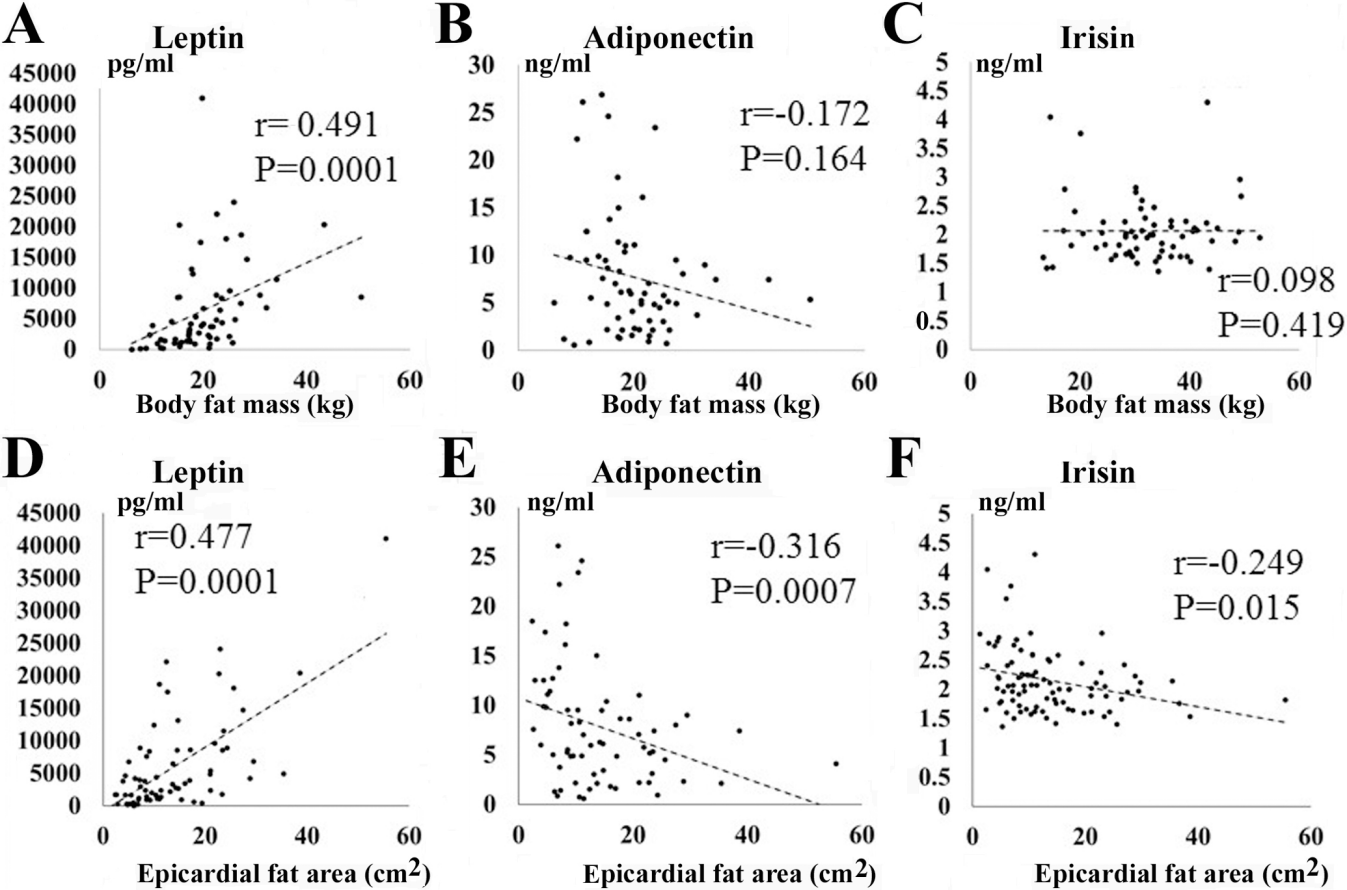
Correlations between serum irisin and adipokines (adiponectin, leptin) level and the findings of BIA method or CT scan. A-C: Relationships between body fat mass and serum irisin and adipokines level (leptin (A), adiponectin (B), and irisin (C)). D-F: Relationships between epicardial fat area (EFA) and serum irisin and adipokines level (leptin (D), adiponectin (E), and irisin (F)).

Table 4 and Fig 4 show the relationships between serum irisin and adipokine (adiponectin, leptin) level and the BIA findings. The serum leptin level was positively correlated with body fat mass (r = 0.491, *P* = 0.0001, Fig 4A) and body fat percentage (r = 0.444, *P* = 0.0001) in the BIA method. In contrast, neither serum adiponectin nor irisin level was correlated with body fat mass (Fig 4B and 4C) or body fat percentage. The serum adiponectin level was negatively correlated with skeletal muscle volume (SMV) and lean body mass (LBM). No relationships between the serum irisin level and these muscle parameters were observed. In addition, no significant relationships between UCG findings (LAD, LVDd, LVDs, IVST, LVPWT, LV mass, EF, and E/A) and serum irisin concentration were observed (data not shown).

Table 4 and Fig 4 also show the relationships between serum irisin and adipokines (adiponectin, leptin, TNF-α) level and the CT scan data. The serum TNF-α level was not significantly correlated with EFA (r = 0.035, *P* = 0.722), VFA (r = -0.111, *P* = 0.380), and SFA (r = -0.216, *P* = 0.083). On the other hand, the serum leptin level was positively correlated with EFA (r = 0.477, *P* = 0.0001, Fig 4D), VFA (r = 0.416, *P* = 0.001), and SFA (r = 0.485, *P* = 0.0001). In contrast, the serum adiponectin level was significantly negatively correlated with EFA (r = -0.316, *P* = 0.0007, Fig 4E), VFA (r = -0.430, *P* = 0.0001), and SFA (r = -0.412, *P* = 0.001). The serum irisin level was also negatively correlated with EFA (r = -0.249, *P* = 0.015, Fig 4F), and SFA (r = -0.223, *P* = 0.039), and tended to correlate with VFA (r = -0.198, *P* = 0.067).

### Relationships among the circulating irisin, adiponectin, and leptin concentrations

Table 5 shows the relationships among serum irisin, adiponectin, and leptin levels. The serum level of adiponectin was negatively correlated with that of leptin in all subjects (r = -0.296, *P* = 0.012) and in females (r = -0.561, *P* = 0.001), but not in males (r = -0.257, *P* = 0.105). However, there were no significant correlations between irisin and adiponectin or leptin in all subjects, males, or females.

**Table 5:** Relationships between various serum adipokine level.

Total patients	*r*-value	*P-*value
Irisin / adiponectin	0.097	0.420
Irisin / leptin	0.023	0.847
Adiponectin / leptin	**-0.296**	0.012*
Male patients	*r-*value	*P*-value
Irisin / adiponectin	0.123	0.444
Irisin / leptin	0.038	0.809
Adiponectin / leptin	-0.257	0.105
Female patients	*r-*value	*P*-value
Irisin / adiponectin	0.017	0.927
Irisin / leptin	-0.005	0.979
Adiponectin / leptin	**-0.561**	0.001**

*P<0.05

**P<0.01

### Multiple regression analysis between serum leptin, irisin and adiponectin level and EFA

The correlations between serum leptin, irisin and adiponectin level and EFA are shown in Table 6. Univariate correlation analysis showed a positive correlation between log (serum leptin concentration) and log (EFA) (β = 0.542, *P* = 0.0001). On the other hand, a negative correlation was observed in between log (serum adiponectin concentration) and log EFA (β = -0.288, *P* = 0.012). Similarly, a negative correlation was observed in between log (serum irisin concentration) and log (EFA) (β = -0.314, *P* = 0.006). In multiple regression analysis, log (EFA) showed a positive association with log (serum leptin level) (β = 0.438, *P* = 0.0001) and a negative correlation with log (serum adiponectin level) (β = -0.260, *P* = 0.015), and log (serum irisin level) (β = -0.204, *P* = 0.038) after adjusting for age, sex, and BMI.

**Table 6:** Multiple linear regression analysis of epicardial fat area (EFA) and adipokines.

	Dependent variable: Epicardial fat area (EFA) (log)		
	Model 1*	Model 2*	Model 3*
Independent variable.	β-value (*P*)	β-value (*P*)	β-value (*P*)
Leptin (log)	0.542 (0.0001**)	0.614 (0.0001**)	0.438 (0.0001**)
	Dependent variable: Epicardial fat area (EFA) (log)		
	Model 1*	Model 2*	Model 3*
Independent variable.	β-value (*P*)	β-value (*P*)	β-value (*P*)
Adiponectin (log)	-0.288 (0.012*)	-0.334 (0.009**)	-0.260 (0.015*)
Irisin (log)	-0.314(0.006**)	-0.290 (0.013*)	-0.204 (0.038*)

Model 1, unadjusted; Model 2, adjusted by age and sex; Model 3, adjusted by age, sex, and BMI

*P<0.05

**P<0.01

## Discussion

The major findings of the present study are as follows: (1) EFA, SFA, and VFA were determined by CT scans in cardiovascular surgery patients. (2) EFA was strongly correlated with BMI (*P* = 0.0001), non-HDL-C (*P* = 0.029), TG (*P* = 0.004), body fat mass (*P* = 0.0001), and body fat percentage (*P* = 0.0001). (3) Serum leptin concentration showed a statistically significant positive correlation with BMI (*P* = 0.0001), and TG (*P* = 0.0001). Adiponectin, but not irisin, showed a significant negative correlation with BMI (*P* = 0.006), and TG (*P* = 0.001). (4) The serum leptin level had a significant positive correlation with EFA in all the participants (*P* = 0.0001). EFA was negatively correlated with irisin (*P* = 0.015) and adiponectin (*P* = 0.0007). (5) The serum level of adiponectin was negatively correlated with that of leptin (r = -0.296, *P* = 0.012), but there were no significant correlations between irisin and adiponectin or leptin. (6) Multivariate linear regression demonstrated that EFA showed a positive association with serum leptin level (β = 0.438, *P* = 0.0001) and a negative correlation with serum irisin level (β = -0.204, *P* = 0.038) and serum adiponectin level (β = -0.260, *P* = 0.015) after adjusting for age, sex, and BMI. The study has provided the first evidence of associations of serum irisin and adipokines (adiponectin and leptin) with epicardial fat in cardiovascular surgery patients. It is likely that circulating irisin plays a role in preventing excess adiposity, especially in epicardial fat, and subsequently cardiovascular risk in patients.

Adiponectin and leptin are adipose tissue-specific proteins, and secreted from adipose tissue [28,29]. Leptin exhibits pro-inflammatory properties and the concentration of this adipokine is increased in obese subjects [35]. The present study showed that there are significant positive correlations between the serum concentration of leptin and the metabolic risk factors, T-Chol, TG, LDL-C, non-HDL-C, and HOMA-IR, BMI, body fat mass, and body fat percentage. On the other hand, adiponectin is an anti-inflammatory and anti-atherogenic mediator released by adipose tissue. In contrast to leptin, plasma levels of adiponectin are reduced in obesity, hypertension, hyperlipidemia, DM, and coronary atherosclerosis [35–38]. It has also been reported that adiponectin mRNA expression in adipose tissue is decreased in obese ob/ob mice and obese humans [36], and is lower in patients with CAD [39–41]. In the present study, serum adiponectin concentration was inversely correlated with the metabolic risk factors, fasting glucose and TG, while it was positively correlated with HDL-C, suggesting that adiponectin also influences lipid metabolism [32,42,43]. The present study demonstrates that serum adiponectin concentration was inversely correlated with leptin concentration in female patients undergoing cardiovascular surgery. This is compatible with a previous paper in normal-weight and obese women [44].

The FNDC5 gene encodes a type I membrane protein that is processed proteolytically to form a new hormone secreted into blood, termed irisin. Irisin is a novel hormone secreted by myocytes (myokines) that has been proposed to mediate the beneficial effects of exercise on metabolism [15,16]. It has been reported to induce the transformation of white adipocytes into beige / brite adipocytes similar to brown adipocytes, and then thermogenesis to increase energy expenditure in mouse [15,16] and human adipocytes [17,18], which suggests that it exerts anti-obesity and anti-diabetic effects [19,20]. However, studies on the association between irisin and metabolic risk factors have shown conflicting results. Choi et al. [45] reported that serum irisin level was significantly negatively correlated with 2-hour plasma glucose, HbA1c, and TG in new-onset type 2 diabetes. Huh et al. [46] found that circulating irisin level was inversely correlated with T-Chol, and HDL-C in middle-aged healthy women and obese subjects, while irisin was positively associated with HDL-C in patients with chronic kidney disease [47]. In addition, only HOMA-IR was an independent factor in a study of Elbert et al. [48], but Lee at al. [49] did not show a significant association with facets of the metabolic syndrome, including fasting glucose and lipid profile, in PD patients. We have also previously reported that HOMA-IR was an independent variable associated with circulating irisin concentration in Japanese obese patients (BMI 36.5 ± 4.7 kg/m^2^) [27]. These discrepancies may be due to the different populations of patients or subjects studied (sex, age, BMI, and types of diseases). The present study failed to show a significant association with fasting glucose, lipid profiles (TG, T-Chol, LDL-C, non HDL-C), or HOMA-IR in cardiovascular patients (BMI 24.1 ± 4.8 kg/m^2^). Excessive accumulation of adipose tissue is a significant source of reactive oxygen species (ROS) and pro-inflammatory cytokines such as TNF-α, resulting in LV dysfunction, increased fibrosis and decreased contractility [50–52]. Especially, EAT is a major source of inflammatory cytokines including TNF-α and ROS, which may contribute to cardiac remodeling [53]. However, we failed to find any correlations between EFA, VFA, and SFA and serum d-ROMs level or TNF-α concentration in cardiovascular surgery patients. But, the further detailed studies using a larger number of patients are required to investigate the relationships between cardiovascular risk factors and circulating irisin in cardiovascular patients.

We also showed that adiponectin was negatively correlated with the serum BNP level. The association of serum adiponectin level with the NYHA class and BNP levels has been reported in chronic heart failure (CHF) [54,55]. Circulating adiponectin concentration was also inversely correlated with skeletal muscle volume (SMV) and lean body mass (LVM), as shown in Table 4, suggesting that adiponectin may play a role in the pathogenesis of cachexia [55]. Irisin is a novel hormone secreted by myocytes including cardiac myocytes. It has been reported that both aerobic and resistance exercise increase circulating irisin in humans [21–23]. In healthy women, circulating irisin had a positive association with biceps circumference used a surrogate marker of muscle mass [46]. Stengel et al. [56] also showed that circulating irisin concentration was positively correlated with fat-free mass using a BIA method in patients with anorexia nervosa (BMI 12.6 ± 0.7 kg/m^2^), normal weight controls (BMI 22.6 ± 0.9 kg/m^2^), and obese patients (BMI 30–40, 40–50 and >50 kg/m^2^). In the present study, there were no significant relationships between irisin concentration and SMV, LVM and UCG parameters including LV mass (data not shown) in cardiovascular patients (BMI 24.1 ± 4.8 kg/m^2^). However, the present study provided the first evidence of associations of serum irisin and adipokines (adiponectin and leptin) with epicardial fat in cardiovascular surgery patients.

The present study showed that the serum leptin concentration was significantly lower in males than in females, and it had a significant positive correlation with the EFA, VFA, SFA as well as BMI, body fat mass, and body fat percentage. Multivariate linear regression demonstrated that EFA had a positive association with serum leptin (β = 0.438, *P* = 0.0001) after adjusting for age, sex, and BMI. Lima-Martinez et al. [12] reported an inverse relationship between EAT thickness and plasma adiponectin concentration in metabolic syndrome patients. Similarly, an inverse relationship between adiponectin and visceral adipose volume measured by CT has been reported in women [13]. However, Harada et al. [14] failed to find a significant association between epicardial fat volume and plasma adiponectin in non-obese patients suspected of having coronary artery disease. In the present study, the serum adiponectin level was inversely correlated with EFA, VFA, and SFA, as well as BMI. In multivariate linear regression analysis, EFA showed a significant negative correlation with serum adiponectin after adjusting for age, sex, and BMI (β = -0.260, *P* = 0.015). In addition, we found for the first time that serum irisin level was negatively correlated with EFA (r = -0.249, *P* = 0.015), and SFA (r = -0.223, *P* = 0.039), and tended to correlate with VFA (r = -0.198, *P* = 0.067). Multivariate linear regression demonstrated that EFA showed a negative correlation with serum irisin (β = -0.204, *P* = 0.038) after adjusting for age, sex, and BMI. Huh et al. [46] reported that circulating irisin was negatively correlated with adiponectin in middle-aged women and obese subjects. However, in the present study, there were no significant correlations between irisin and either adiponectin or leptin.

Irisin has been reported to cause the transformation of white adipocytes into beige / brite adipocytes, white adipocytes with a phenotype similar to brown adipocytes, and then to increase thermogenesis by increasing uncoupling protein 1 (UCP1) levels, thereby increasing energy expenditure, in mice [15–18]. Epicardial fat has relatively abundant UCP-1 expression, compared with visceral and subcutaneous fat, and is a characteristic of beige adipocytes, white adipocytes with a phenotype similar to brown adipocytes [3,57]. It remains unclear whether circulating irisin can increase UCP-1 expression and enhance brown adipose tissues, and further studies are needed to clarify the effects of irisin on adipose tissues in cardiovascular patients. However, from these results, it is likely that serum irisin concentration may play a role in preventing excess adiposity, especially epicardial fat, and then cardiovascular risk in patients. Exercises including endurance [21], aerobic training combined with resistance training [22], and resistance training alone [23], have been reported to increase circulating irisin and decrease fat mass in healthy and obese subjects. Therefore, further interventional studies using cardiovascular patients such as exercise and diet therapy on fat and serum irisin concentration are required to clarify these possibilities.

Some limitations of our study need consideration. First, because it was a cross-sectional study, the results did not imply causality. Second, the study had a small number of patients undergoing different types of cardiovascular surgery (i.e. CABG, and valve repair/replacement) and there were no control subjects. In addition, since most of the subjects had medical treatment, such as statins and diabetic drugs, our data on risk factors, including the lipid profile, may reflect the effects of medications to some extent. Thus, our findings are not necessarily applicable to the general population. Third, we used the epicardial fat area determined by a plane image instead of measuring the volume of epicardial fat. Therefore, further detailed analyses in a large number of patients and interventional studies, such as exercise and diet therapy, of epicardial and serum irisin concentrations are required to clarify our findings.

## Conclusions

EFA measured by CT scan was positively correlated with leptin (*P* = 0.0001) and negatively correlated with irisin (*P* = 0.015) and adiponectin (*P* = 0.0007). Multivariate linear regression demonstrated that EFA showed a positive association with serum leptin level (β = 0.438, *P* = 0.0001) and a negative correlation with serum irisin level (β = -0.204, *P* = 0.038) and serum adiponectin level (β = -0.260, *P* = 0.015) after adjusting for age, sex, and BMI. These results suggest that irisin may play a role in preventing excess adiposity, and reducing cardiovascular risk in patients.
